# Management of Severe Traumatic Brain Injury in Pregnancy: A Body with Two Lives

**DOI:** 10.21315/mjms2018.25.5.14

**Published:** 2018-10-30

**Authors:** Giat Seng Kho, Jafri Malin Abdullah

**Affiliations:** 1 Department of Neurosciences, School of Medical Sciences, Universiti Sains Malaysia, 16150 Kubang Kerian, Kelantan, Malaysia; 2 Centre for Neuroscience Services and Research, Universiti Sains Malaysia, 16150 Kubang Kerian, Kelantan, Malaysia; 3 Department of Neurosurgery, Sarawak General Hospital, Jalan Hospital, 93586 Kuching, Sarawak, Malaysia

**Keywords:** severe, traumatic brain injury, brain, pregnancy, management

## Abstract

Traumatic brain injury is the major contributing factor in non-obstetric mortality in developing countries. Approximately 20% of maternal mortality is directly correlated to injuries. Road traffic accidents and domestic violence are the most common nonlethal injuries that can threaten either the maternal or foetal life, and such events occur in one out of every 12 pregnancies. The treatment of severe traumatic brain injury in pregnancy requires a multidisciplinary team approach. The management of a pregnant trauma patient warrants consideration of several issues specific to pregnancy, such as the alterations in the maternal physiology and anatomy. In the case of maternal cardiac arrest with amniotic fluid embolism, intact neonatal survival is linked with the timing of caesarean section after maternal cardiac arrest. Moreover, the decision for perimortem caesarean section is clear after maternal cardiac arrest. The foetal survival rate is 67% if the operation is done before 15 min of cardiopulmonary compromise has occurred, and it drops to 40% at the duration range of 16–25 min. Whether minor or severe, traumatic brain injury during pregnancy is associated with unfavourable maternal outcomes. Injuries considered minor for the general population are not minor for pregnant women. Therefore, these patients should be intensively monitored, and multidisciplinary approaches should always be involved.

## Introduction

Traumatic brain injury is the major contributing factor in non-obstetric mortality in developing countries. Approximately 20% of maternal mortality is directly correlated to such injuries. Road traffic accidents and domestic violence are the most common nonlethal injuries that can threaten either the maternal or foetal life, and such events occur in 1 out of every 12 pregnancies (1, 2).

The physiological adaptations that occur as pregnancy progresses warrant several specific considerations in the management of severe traumatic brain injury in pregnant patients. Aside from maternal injuries, foetal wellbeing and conditions that are unique to pregnancy or related to trauma (Rh immunisation, miscarriage, preterm labour, placental abruption and foetal demise), clinicians must keep in mind the possible radiation exposure and other teratogens (2). This review focusses on the evidence-based management of severe traumatic brain injury in pregnancy, from pre-hospital to post-operative care.

### Pre-hospital Care

Paramedics should always presume that every female of reproductive age with significant injuries is pregnant until proven otherwise by a definite pregnancy test or ultrasound scan (III-C) (3). Oxygen supplementation is essential for both the mother and foetus for the prevention of hypoxia. Pregnancy should prompt positioning the patient in the left or right lateral decubitus position or manual displacement of the uterus to avoid compression of the vena cava, resulting in hypotension. Hypoxia and hypotension have detrimental effects on both lives, as these conditions are associated with approximately 50%–75% of mortalities. In general, treatment that is good for the mother is good for the foetus. For patients with a suspected spine fracture, the left lateral tilt position can be utilised.

## In the Emergency Room

A complete obstetric and neurosurgical history is mandatory, as this will influence decision making. In cases of major trauma, the assessment, stabilisation and care of the pregnant women is the first priority; then, if the foetus is viable (≥ 23 weeks), foetal heart rate auscultation and foetal monitoring can be initiated, and an obstetrical consultation should be obtained as soon as is feasible (II-3B) (3).

In pregnant women with a viable foetus (≥ 23 weeks) and suspected uterine contractions, placental abruption or traumatic uterine rupture, urgent obstetrical consultation is recommended (II-3B) (3). In cases of vaginal bleeding at or after 23 weeks, speculum or digital vaginal examination should be deferred until placenta previa is excluded by a prior or current ultrasound scan (III-C) (3).

In view of the narrow range of blood pressure (BP) control, at 140/90 (mild preeclampsia) to 160/110 (severe pre-eclampsia), as well as avoidance of hypotension, BP should be reduced or controlled, aiming for a level of approximately 140/90 mm Hg. O-negative blood should be used if needed, in view of Rh immunisation.

## Radiation Exposure

Diagnostic imaging in injured pregnant women is always delayed in view of reluctance to expose the foetus to ionising radiation. However, this discomfort should be avoided in critical decision making, and the risk of teratogenic potential should be well understood.

- Radiographic studies indicated for maternal evaluation, including abdominal computed tomography, should not be deferred or delayed due to concerns regarding foetal exposure to radiation (II-2B); and
- Use of gadolinium-based contrast agents can be considered when the maternal benefit outweighs potential foetal risks (III-C).

Ionising radiation has the highest teratogenic potential during the period of organogenesis (5–10 weeks), with an increased risk of miscarriage before this period. After 10 weeks, radiation is more likely to produce growth restriction than teratogenic changes. Radiation exposure with a cumulative dose of > 5–10 rad (50–100 mGy) is associated with an increased risk of foetal malformation, usually limited to a gestational age < 18 weeks. There is a less than 6% chance of developing severe mental retardation, less than 3% chance of childhood cancer and approximately 15% chance of microcephaly with the total exposure radiation dose of 15 rad (4, 5), plain chest X-ray generally below 0.005 rad, pelvic X-ray below 0.4 rad and computed tomography of the head (1 cm slices) 0.05 rad (6). Magnetic resonance imaging and ultrasonography have not been associated with any adverse foetal effects. There is no evidence of teratogenic effects with gadolinium administration (3).

## Management for the First and Second Trimesters

In any pregnancy before 13 weeks of gestation, the uterus is protected by the bony pelvis (3). Foetal loss in the first trimester is less likely the result of direct trauma (occurring less than 1% of the time), and instead, it is likely to be caused by uterine hypoperfusion resulting from maternal hypotension or death (7).

Any pregnancy is considered viable after 23 weeks of gestation in view of the low survival rate of the foetus and approximately 61% foetal loss in women with major injuries before this time. The standard guidelines for the management of traumatic brain injury can be applied to pregnant patients with appropriate modifications for the population (8). Prophylactic anti-epileptic therapy can be applied as a pre-emptive measure against intracranial hypertension–induced seizures. If neurosurgical intervention has been performed in early pregnancy (< 24 weeks), the decision about subsequent foetal management can be based on obstetric considerations (7).

## Management for the Third Trimester

Based on the Brain Trauma Foundation 2016 guidelines, a variety of measures to control intracranial pressure can be administered, such as a slight head-up position, low tidal volumes during intermittent positive pressure ventilation and avoidance of vomiting (8). Application of mannitol in pregnant women should involve caution, as it slowly accumulates in the foetus, causing foetal hyperosmolality; this will lead to physiological changes, such as reduced foetal lung fluid production, reduced urinary blood flow and increased plasma sodium concentrations (9, 10). However, in individual case reports, mannitol in doses of 0.25–0.5 mg/kg has been used and appears safe (11, 12). Furosemide is an alternative, but it should also be used cautiously.

## Neurosurgical Intervention

If the foetus is viable (> 24 weeks of gestation) at the time of the planned neurosurgery, a decision must also be made concerning whether delivery is appropriate. Neurosurgeons may face one of the following scenarios:

– Caesarean delivery alone or followed by neurosurgery (simultaneous)
  - When the uterus interferes in trauma-related surgical interventions or
  - With foetal compromise in a viable foetus with a stable mother: placental abruption/uterine rupture (maternal mortality rate of up to 10% and nearly universal foetal mortality); and
– Neurosurgery followed by caesarean/vaginal delivery
  - If cardiopulmonary resuscitation has been unsuccessful after 4 min or there is obvious impending or recent maternal death (III-B) (3).

The chance for maternal survival after cardiopulmonary arrest, as compared with that of women in the non-pregnant state, is only 30% (13). The decision to perform perimortem caesarean section must be made after maternal cardiac arrest, as 67% of foetuses survive if the operation is done before 15 min of cardiopulmonary compromise, but this proportion drops to 40% at 16–25 min (13, 14). According to Katz et al., perimortem caesarean section is best done before 5 min of maternal arrest, as this results in the highest foetal survival rate (15).

## Post-operative Management

Continuing care in the intensive unit for pregnant women involves maintaining neurological and systemic homeostasis; in addition, sustaining the foetal viability should be undertaken (with or without surgery). Good analgesia support should be ensured to facilitate maternal mobility, as well as providing pain relief and avoiding undesirable haemodynamic compromises. Morphine, codeine and tramadol are safely applicable, with few side effects and the best pain control. It is important to avoid cyclo-oxygenase inhibitors, in view of their effects on platelet function and potential bleeding after intracranial surgery and the potential foetal complications that can arise (renal failure, necrotising enterocolitis and persistent foetal circulation after birth), especially after 32 weeks of gestation (16).

There is an increased risk of both deep venous thrombosis and pulmonary embolism due to alterations in clotting factors in pregnancy, as well as endothelial injury secondary to trauma and susceptibility to infection (17). Pharmacological and nonpharmacological prophylaxis measurements should be encouraged, but the risk–benefit analysis of anti-coagulants should be considered after discussion with the neurosurgeon.

## Special Considerations

### Obstetric Complications in Trauma

Management of suspected placental abruption should not be delayed pending confirmation by ultrasonography, as ultrasound is not a sensitive tool for its diagnosis (II-3D) (3). Pregnant patients with severe head injuries are at an increased risk of foetal death. The most common cause of foetal death in trauma patients is placental abruption (18).

### Temperature

The foetal temperature parallels the maternal temperature, and both maternal hyperthermia and hypothermia may be associated with increased morbidity in the presence of increased intracranial pressure (19). Preservation of the normal body temperature of the pregnant patient undergoing neurosurgery may be achieved with a forced air warmer, and the body temperature can be monitored with a urinary bladder or oesophageal temperature probe (20).

### Spine Trauma

Spine trauma in pregnancy should be treated in the same way as in non-pregnant women. Appropriate abdominal shielding for diagnostic imaging can be applied. The prone position for spinal surgery in pregnancy may cause difficulties with respect to foetal monitoring, emergent caesarean delivery and increased epidural venous bleeding (21). However, with the prone position, the placental perfusion may be increased, as reported in Nakai et al. (22).

## Conclusion

**Figure 1:**
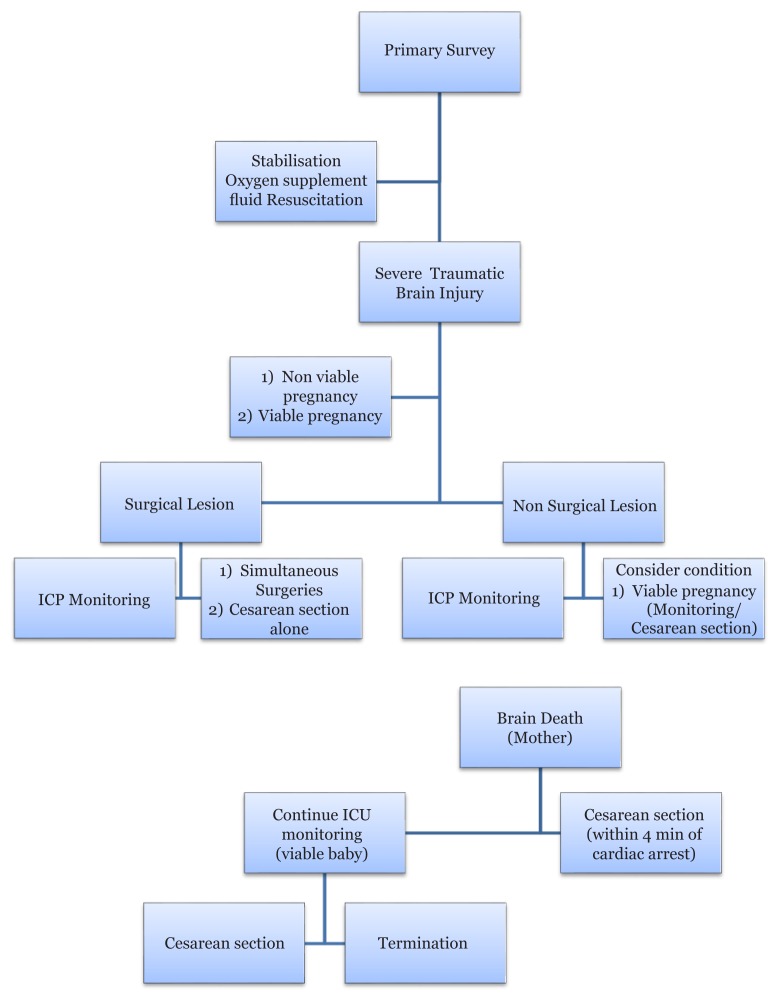
Algorithm management of severe traumatic brain injury in pregnancy, including surgical and nonsurgical intervention as a guideline for medical personnel and hospital facilities, when facing the dilemmas shown

Traumatic brain injury during pregnancy, whether minor or severe, is associated with unfavourable maternal outcomes. Injuries considered minor for the general population are not minor for pregnant women. Therefore, these patients should be intensively monitored, and multidisciplinary approaches should always be involved (Figure 1, Table 1).

## Figures and Tables

**Table 1:** General management of severe traumatic brain injury in mother and baby

	Prehospital Care		Emergency department		OT/ICU		Post-operative	
			
	1st & 2nd	3rd & Labour	1st & 2nd	3rd & Labour	1st & 2nd	3rd & Labour	1st & 2nd	3rd & Labour
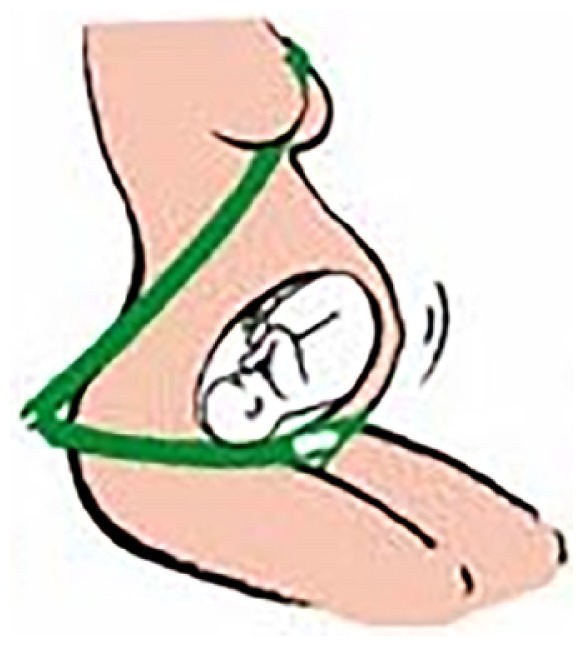	Manage via trauma life support protocol Oxygen supplementation with SPO_2_ > 95% Vasopressor only for intractable hypotension	Gravid uterus should be moved off for better venous return Emergency transfer to the nearest trauma unit for major injury evaluation Avoid anti-shock trouser	Manage via trauma life support protocol Avoid rhesus D alloimmunisation O-negative blood transfusion if needed	Investigation of suspected placental abruption should not be delayed; urgent obstetrical consultation is recommended Imaging as indicated (low radiation exposure & low teratogenic effect)	Mother always the priority in view of 82% foetal deaths in these incidents Surgical intervention as needed: ICP monitoring Craniotomy Termination	Simultaneous craniotomy+ C-section Maternal brain death: Continue ICU support	Monitoring following Traumatic Brain Foundation 2016: Euvolaemia Keep normotensive ICP monitoring GCS & pupil monitoring	Nonsurgical lesion ICP monitoring Continue foetal wellbeing monitoring Prepare for delivery with O&G team standing by Surgical lesion (as simultaneous procedures) Manage following Traumatic brain foundation 2016
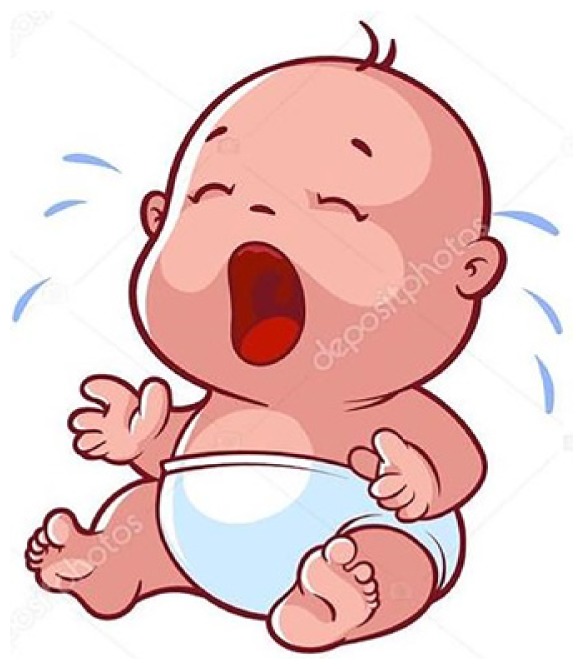	Pregnancy before 24 weeks of gestation is considered nonviable	Inform call centre for multidisciplinary team management (Neurosurg, O&G, Surg, Paeds & Radio)	Pregnancy below 24 weeks of gestation is considered nonviable Refer O&G for the mode of management.	Electrical foetal monitoring for at least 4 h Urgent obstetric ultrasonic scan Foetal wellbeing, carefully documented for legal purposes	Advice for spontaneous delivery rather than surgical intervention in view of nonviable foetus	Maternal brain death No later than 4 min, subject for C-section	N/A	Admit under NICU

Overall management of the mother and baby in the case of severe traumatic brain injury in the mother, throughout the separate partum and post-partum periods, is shown. There is controversy regarding the treatment options for a viable baby. Duration of foetal monitoring following a traumatic event falls toward the ability to identify potential trauma-related foetal problems and premature labour or foetal distress. Uterine contraction is the most common traumata-related consequence, with secondary induction of increased intracranial pressure, with the potential for endangering both the mother and baby; necessary management should be instituted as soon as possible, without interfering with maternal resuscitation efforts
